# Death of Dr. Abercrombie

**Published:** 1845-01

**Authors:** 


*Death of Dr. Abercrombie.*—It is with great regret that we announce to the profession the death of Dr. John Abercombie, which took place, suddenly, at his house, York-place, Edinburgh, on Thursday morning, November 14th.

Dr. Abercrombie was the son of the late Rev. Mr. Abercrombie, one of the ministers of Aberdeen. He took his degree at Edinburgh on the 4th of June, 1803, writing for his Thesis, "*De fatuitate Alpina.*" After studying in London for six months, he became a fellow of the Royal College of Surgeons, Edinburgh, and settled in that city. At this period Drs. Gregory and Munro Saunders were in full practice as consulting physicians. Dr. Abercrombie commenced as a general practitioner, but from the first he succeeded in gaining, to a remarkable extent, the confidence of the public. His success is said to be owing to the assiduous attention he paid his patients. This he carried to an extent previously unknown, frequently visiting them three or four times a day. His unusual success created many rivals and enemies: these he disarmed, and even ultimately converted into friends, by the inoffensiveness of his conduct, and frequently by practising the Christian doctrine he professed, of returning good for evil. In 1808 he married a lady of considerable fortune, which enabled him to keep his carriage. In 1806 he made his first communication to our respected contemporary, the *Edinburgh Medical and Surgical Journal*, entitled, "A Case of Cynanche Laryngea." It is inserted in the twelfth volume of that periodical. In the pages of its subsequent numbers he became a frequent contributor, and they will be found to contain most of the cases and observations, which he afterwards embodied in his well-known works on the Pathology of the Brain and Abdominal Organs.

On the death of Dr. Gregory, in 1821, he became a candidate for the chair of physic, held by that distinguished physician. Dr. James Home, however, at that time very popular as a professor of materia medica, was translated to the chair of physic, and was succeeded by the late Dr. Duncan. Dr. Abercrombie now joined the Royal College of Physicians, and gradually became the first consulting physician in the Scottish metropolis. In this capacity he acquired an extent of practice and public confidence which distanced all competitors.

Notwithstanding the harassing nature of his avocations, the contributions of Dr. Abercrombie to medical literature were numerous and important. In 1820 appeared his *Researches on the Pathology of the Intestinal Canal*. In 1828 was published his celebrated work, entitled, *On Diseases of the Brain and Spinal Cord*. It has been translated into most of the European languages, and has gone through three English editions. This was followed by an enlargement of his work *On the Intestinal Canal*, called *On Diseases of the Abdominal Viscera*, of which a second edition was required in 1830. In this year also he gave the public a book *On the Intellectual Powers and Investigation of Truth*, of which popular work several editions have been published. This was followed, in 1832, by *Suggestions on the Malignant Cholera*, and in 1833, by *The Philosophy of the Moral Feelings*.

In 1835 Dr. Abercrombie was elected lord rector of Marêchal College and University, Aberdeen, and published his inaugural Address, which afterwards appeared in an enlarged form, under the title of "Culture and Discipline of the Mind." In 1834 the University of Oxford conferred upon him the degree of M. D., and he was elected one of the vice-presidents of the Royal Society of Edinburgh.

In 1841 Dr. Abercrombie had an attack of paralysis, from which, however, he soon recovered, and resumed the active duties of his profession. His health remained good up to the moment of his decease. On Thursday, Nov. 14, 1844, after eating a hearty breakfast, he was preparing to go out, as usual. The carriage was at the door, but as he remained longer than usual, a servant entered his private room, and found him lying on his face, dead.

On the following Saturday a *post-mortem* examination revealed the following facts :—The brain was very large, weighing forty-six ounces, but healthy in structure throughout. The pericardium was distended with blood, which had been poured out from a laceration in one of the branches of the coronary artery, about four lines in length.

In reviewing the reputation of Dr. Abercrombie as an author, we cannot but feel surprised that he should have attained so eminent a scientific position, independent of those means which with other men are necessary to its acquirement. He was never physician to any public hospital or dispensary, and all the facts with which his works are enriched were derived from private practice. Yet his treatises on the Brain and Abdominal Organs embody a mass of the most interesting cases and observations, and constitute most important contributions to the subjects of which they treat. Moreover, they tended to maintain and extend the scientific claims of British physicians, and this notwithstanding the *éclat* deservedly enjoyed by the pathologists of France. His philosophical work, *On the Intellectual Powers*, &c., may still be considered the most interesting and lucid introduction to a study of the mental faculties, although it emanates from the same school which boasts of Dugald Stewart, of Reid, and of Brown. In his latter years, Dr. Abercrombie, by his writings, extended, in no small degree, a knowledge of the principles of revealed religion. The short essays he published on sacred truths have been most extensively read, from twenty to thirty thousand copies of each having been sold in a few years.

The private life and conduct of Dr. Abercrombie were not only irreproachable, but distinguished by the utmost philanthropy and good will towards all men. In him all the public charities of Edinburgh have lost a supporter, and many are the instances which might be related of the assistance he has rendered his professional brethren when in distress. Amongst others may be mentioned the case of a general practitioner, who, from a train of adverse circumstances, being declared bankrupt, received from Dr. Abercrombie the sum of one hundred guineas, on the day his effects were sold.

The confidence he enjoyed amongst the profession was deservedly great. He never attempted to supplant a brother practitioner, or ingratiate himself with the public by means unworthy the dignity of the physician. His manner towards a patient, indeed, was characterized by great taciturnity, although never by rudeness. All his appointments were kept with scrupulous exactitude, and to this, as well as the gentlemanly conduct he pursued in his consultations with the profession, must be attributed much of the extensive confidence they placed in him up to the moment of his death.—*London Lancet.*

